# Trait Variation and Spatiotemporal Dynamics across Avian Secondary Contact Zones

**DOI:** 10.3390/biology13080643

**Published:** 2024-08-22

**Authors:** Shangyu Wang, Lei Wu, Qianghui Zhu, Jiahao Wu, Shiyu Tang, Yifang Zhao, Yalin Cheng, Dezhi Zhang, Gexia Qiao, Runzhi Zhang, Fumin Lei

**Affiliations:** 1 Key Laboratory of Zoological Systematics and Evolution, Institute of Zoology, Chinese Academy of Sciences, Beijing 100101, China; wangshangyu21@ioz.ac.cn (S.W.); wulei@ioz.ac.cn (L.W.); zhuqianghui@ioz.ac.cn (Q.Z.); tangshiyu@ioz.ac.cn (S.T.); zhaoyifang21@mails.ucas.ac.cn (Y.Z.); zhangdezhi@ioz.ac.cn (D.Z.); zhangrz@ioz.ac.cn (R.Z.); 2 University of Chinese Academy of Sciences, Beijing 100049, China; wujiahao20@mails.ucas.ac.cn; 3 Guangdong Public Laboratory of Wild Animal Conservation and Utilization, Guangdong Key Laboratory of Animal Conservation and Resource Utilization, Institute of Zoology, Guangdong Academy of Sciences, Guangzhou 510260, China; 4 College of Life Sciences, Institute of Life Science and Green Development, Hebei University, Baoding 071002, China; chengyalin001@163.com

**Keywords:** secondary contact zone, avian, speciation, trait variation, spatiotemporal dynamics

## Abstract

**Simple Summary:**

This review aimed to provide an exhaustive overview of the advancements in avian SCZ research. In the review, we summarize the latest research on trait variations in avian SCZs, including vocalization, plumage, beak, and migratory traits. In addition, we discuss the mechanisms of different types of avian SCZ movements. Finally, we outline several significant questions for future studies.

**Abstract:**

A secondary contact zone (SCZ) is an area where incipient species or divergent populations may meet, mate, and hybridize. Due to the diverse patterns of interspecific hybridization, SCZs function as field labs for illuminating the on-going evolutionary processes of speciation and the establishment of reproductive isolation. Interspecific hybridization is widely present in avian populations, making them an ideal system for SCZ studies. This review exhaustively summarizes the variations in unique traits within avian SCZs (vocalization, plumage, beak, and migratory traits) and the various movement patterns of SCZs observed in previous publications. It also highlights several potential future research directions in the genomic era, such as the relationship between phenotypic and genomic differentiation in SCZs, the genomic basis of trait differentiation, SCZs shared by multiple species, and accurate predictive models for forecasting future movements under climate change and human disturbances. This review aims to provide a more comprehensive understanding of speciation processes and offers a theoretical foundation for species conservation.

## 1. Introduction

Secondary contact refers to a scenario in which gene flow occurs between genetically distinct populations [1]. A secondary contact zone (SCZ) is an area where these populations may meet, mate, and hybridize during secondary contact [2]. It is widely accepted that the formation of SCZs is closely associated with both past and present climate changes [3]. In particular, during the Pleistocene glacial period, suitable habitats for species were contracted due to the expanded ice sheets and colder temperatures [4]. Refugia were found in more suitable places. As the climate changed due to global warming at the end of the last glacial maximum (LGM), the ice cover on the continents melted, providing new land for habitats [5]. This led to a rapid expansion of species from glacial refugia and populations reconnected, resulting in the formation of SCZs [6,7,8,9,10]. In recent years, the role of anthropogenic disturbances, including deforestation and urbanization, has gained recognition as a significant factor in the formation of SCZs. This is primarily attributed to habitat loss and modification, the creation of artificial corridors, and the introduction of exotic species [11,12].

SCZs are often regarded as the “window of the evolutionary process” because they offer opportunities to study trait variations in individuals with mixed genetic backgrounds and to quantify gene flow across different genomic regions [13]. The patterns of gene flow are determined by the biogeographical structure, the gene introgression pattern, and the species-specific characteristics [14]. There are three main factors affecting secondary contact and natural selection in SCZs, including population structure diversification, interspecies accessibility, and the fitness of hybrids [2]. Consequently, hybridization between different taxa may lead to different outcomes. Depending on the patterns of interspecific gene flow, secondary contact may result in various consequences for populations, such as continuous hybrid zones [15], hybrid speciation [16], reverse speciation [17], and extinction [18,19]. Continuous intrinsic and extrinsic changes may lead to dynamic changes in the genetic structure and range shifts of populations over time [20]. The movements of SCZs have significant consequences for both evolutionary and conservation biology because they can enhance our understanding of how past and current selection pressures affect the structure and distribution of these zones [21]. In particular, under climate change and human influence, the movements will provide important references for studying interspecies interactions, environmental adaptation, and the conservation of endangered species [22].

Advances in genome sequencing technology have revolutionized population genetics, particularly population genomics, ushering in a new era in this field. Early studies on SCZs relied heavily on field exploration, museum specimen records, and hybrid experiments [23,24]. The degree of hybridization was quantitatively represented by calculating the hybrid index [25]. Molecular biology advancements, such as electrophoretic separation and PCR technology, have facilitated SCZ research [26,27]. First-generation molecular marker technologies, like restriction fragment length polymorphisms (RFLPs), provided insights into genetic structure and evolutionary history [28]. Second-generation markers, including random amplified polymorphic DNA (RAPD) [29], amplified fragment length polymorphisms (AFLPs) [30], simple sequence repeats (SSRs) [31], and mitochondrial DNA markers (such as mitochondrial protein-coding genes [32] and complete mitochondrial genomes) [33], offered high-resolution genetic information for detecting SCZs [34,35]. Over the past decade, third-generation single-nucleotide polymorphism (SNP) markers have gained popularity for their robustness and broad genomic distribution, facilitating rapid screening and addressing phylogenetic, taxonomic, and hybridization inquiries [36,37]. Restriction-site-associated DNA sequencing (RAD-seq) has emerged as a cost-effective, accurate, and efficient approach that is applicable to nonmodal species without reference genomes [38]. With the publication of a large number of high-quality avian reference genomes [39], whole-genome resequencing has been widely used for research on avian population genetics [40], phylogeny [41], and important economic traits [42,43]. These technologies have shed light on genomic variations, spatial patterns, the impact of secondary contact on the genome [44,45], and the interaction between hybridization and environmental changes [46,47].

Birds, the most diverse group of land vertebrates, have become a focal point of research across many evolutionary fields, such as research on biogeography [48,49,50], phylogenetics [41,51,52,53], adaptation [54,55,56], and speciation [57,58]. Among taxa of comparable diversity, we possess the most thorough knowledge on the species-level taxonomy and geographical distribution of birds. The ease of observing and capturing birds in the wild and obtaining museum specimens has facilitated the collection of valuable information on the heritability and responses to the selection of morphological traits in natural settings [59]. In many avian research systems, traditional studies on geographic distribution, ecology, and reproduction have spanned decades, facilitating research on SCZ movement and the prevalence of research on interspecific hybridization among birds [60]. Avian genomes are relatively small, conserved, and unique among vertebrates in terms of their genome organization [61], making them one of the most densely sampled higher-level animal taxa in genomics research [62]. The first bird genomes sequenced were the economically important chicken [63]; the zebra finch (*Taeniopygia guttata*) [64], a model species for vocal learning; and the ground tit (*Parus humilis*), a species that helped correct traditional taxonomic errors through using genomic approaches [65]. Recently, more colleagues have pursued bird genome and morphology projects, and a growing number of studies have focused on secondary contact among avian clades [66], sister species [67], non-sister species [68], and subspecies [69]. 

Long-term ecological research accumulation and recent advances in genomics have ushered the research on avian SCZs into a new era. Recently, many articles have been published on the trait variations in birds in SCZs and the movement of these zones. However, there are no published review articles that focus on the integration of avian-specific traits in SCZs and the latest research on the movements of avian SCZs. In order to summarize the existing research and explore potential future directions in this field, we wrote this review with the aim of providing an exhaustive overview of the advancements in this field. In this review, we chiefly summarize the latest research on trait variations in avian SCZs, including vocalization, plumage, beak, and migratory traits. In addition, we discuss the mechanisms of avian SCZ movements and give examples of different types of movements. Finally, we outline several significant questions for future studies.

## 2. Trait Divergence in Avian SCZs

SCZs have the potential to give rise to populations with novel adaptive variations or functional traits, contributing to the evolution of phenotypic diversity [70,71]. Studying phenotypic variations in SCZs offers valuable insights into the fundamental mechanisms of reproductive isolation. In this section, we will focus on distinctive traits in birds, including plumage, song, and beak traits, which demonstrate remarkable plasticity within SCZs. The variation in these traits is influenced by both natural and sexual selection and represent species-specific adaptations that play a significant role in avian reproductive isolation and interspecific gene flow [72].

### 2.1. Vocalization

Vocalization is a bioacoustic trait that is important in reproductive and territorial activities [73,74]. In avian SCZs, vocal differentiation may result from natural selection or drift, and contributes to the formation of reproductive isolation [75,76]. Natural selection may reduce the fitness of hybrid individuals, and vocalization may cause further differentiation through the reinforcement of prezygotic reproductive isolation [77,78]. Even in the absence of apparent vocal differences in the contact zone, the ability of vocal recognition may be enhanced [79]. However, in other evolutionary scenarios, vocalizations in the contact zone may undergo assimilation [80,81,82], facilitating hybridization between sympatric populations [83,84]. 

**Figure 1:**
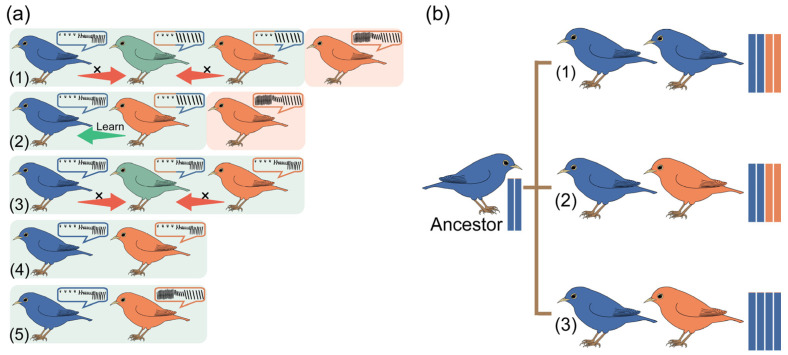
Patterns of song and plumage variations with gene flow. The blue and orange colors in the boxes represent genetic backgrounds. The light green and orange represent distributions. (**a**) Relationship between song divergence, distribution, and genomic variation. (1) The distribution zones overlap, the vocalizations are different, and in the overlapping region, one species learns the song of the other species. Hybridization occurs, and the non-native species only produces a single type of vocalization. (2) The distribution zones overlap, the vocalizations are different, and in the overlapping region, only one species learns the song of the other species, with no hybridization occurring. The non-native species only produces a single type of vocalization. (3) The distribution zones overlap, and the vocalizations are similar, hybridization may occur in the overlapping region, and there may be heterospecific song learning. (4) The distribution zones overlap, and the vocalizations are similar. However, in the overlapping regions, there is no hybridization, and there is no occurrence of heterospecific song learning. (5) The distribution zones overlap, but the vocalizations are different. The overlapping regions do not result in hybridization, nor do the individuals learn the songs of the other species. (**b**) Relationship between plumage divergence and genomic variation. (1) Conspicuous plumage divergence accompanied by a mixed genetic structure. (2) Obvious plumage divergence despite limited genetic divergence. (3) Variation in specific plumage traits resulting from distinct genetic variations at several key loci.

The mixed songs of closely related species are widely observed and may be asymmetric in an SCZ [13,82] (Figure 1a). In some systems, only one of the two species in the contact zone learns the vocalization of the other species, resulting in an asymmetric vocal blending between the two species. For example, in the hybrid zone of the common nightingale (*Luscinia megarhynchos*) and the thrush nightingale (*L. luscinia*) in Europe, individuals producing blended songs were exclusively *L. luscinia* or a few hybrid individuals [85,86]. A similar situation occurred in the contact zone of the collared flycatcher (*Ficedula albicollis*) and the European pied flycatcher *(F. hypoleuca*). Apart from blended-singing hybrid individuals, only *F. hypoleuca* learned the songs of *F. albicollis* [87,88]. In the hybrid zone of the great tit (*Parus major*) and the Japanese tit (*P. minor*) in Russia, individuals producing blended songs were mainly *P. major* and hybrid individuals [89,90]. Similar examples also occurred in the hybrid zones of the Eurasian treecreeper (*Certhia familiaris*) and the short-toed treecreeper (*C. brachydactyla*) [91]. In these cases, it was typically the rarer species that learned the songs of the other species. This suggests that whether a species learns the songs of another species might be related to their relative effective population size. Although mixed songs were found in the SCZ of *Phylloscopus forresti* and *P. kansuensis*, vocal differences still maintained reproductive isolation between the two species and the divergence time was about 2.4 million years [74].

There are many factors that affect vocalization differentiation. Certain “key” elements may play a crucial role in interspecific recognition and information transmission. For instance, playback experiments involving artificially modified song elements showed that *Phylloscopus collybita* displayed a heightened aggressive response to elements with descending modulation, while showing a weaker response to elements with ascending modulation [92]. In some other species experiencing vocal blending and hybridization in contact zones, it has been observed that only individuals within the contact zone exhibit a response to heterospecific vocalizations, while allopatric populations do not, as seen in cases such as *P. collybita abietinus* and *P. collybita tristis* [93]. These findings suggest that the co-occurrence of potential interspecific competitors in contact zones triggers aggressive responses due to resource competition. Souriau et al. (2018) [86] provided evidence for convergent agonistic character displacement in a study of the contact zones of the genus *Luscinia*, wherein interspecific territorial competition appears to be reinforced by convergence in territorial signals [94]. Female preference might be another driving force behind vocal blending in contact zones because of the significant role of sexual selection. Studies on *Parus major* indicate that females prefer more complex and variable songs [95,96], thereby potentially exerting a selective pressure on vocalizations in contact zones, promoting the recombination of repertoires from different species sources [97]. In some species of *Acrocephalus*, the females also exhibited a preference for longer songs, more complex singing, and richer repertoires [98]. However, sexual selection has been suggested to lead to the evolution of short, simple, and stereotyped songs in some other *Acrocephalus* species [99]. Furthermore, differences in vocal learning abilities among species also likely contributed to the asymmetric vocalization. Across avian species, there are differences in vocal learning abilities at broad evolutionary scales [100,101,102]. However, whether closely related species possess similar, or divergent learning abilities remains to be elucidated.

Song is a key signal in avian sexual selection, and therefore vocal blending may also facilitate gene flow between species. The relationship between vocal blending and gene flow varies depending on the complex evolutionary context. In the SCZ of the Iberian chiffchaff (*P. ibericus*) and the common chiffchaff (*P. collybita*), numerous individuals with blended songs were documented, with most individuals exhibiting genotypes that fell between the two species [103]. Approximately 10% of the individuals in the contact zone were derived from F1 hybrids or backcrosses [104]. Similarly, in the contact zone of the *P. collybita abietinus* and *P. collybita tristis* subspecies in Russia, 9.8% of males produced songs that did not match their mitochondrial haplotype [93]. Another study based on whole-genome single-nucleotide polymorphism data found that 11 out of 12 hybrid individuals had mitochondrial DNA and a genomic principal component predominantly derived from *P. collybita tristis*. In Central and Eastern Europe, in an SCZ of *Ficedula albicollis* and *F. hypoleuca*, the vocal blending was found to be unidirectional. Only *F. hypoleuca* fully or partially replicated the song of *F. albicollis* [80,83,88]. In the Swedish contact zone, one study demonstrated that vocal blending facilitated interspecific hybridization [83]. In western Russia, despite approximately 40% of *F. hypoleuca* males being able to produce song elements resembling those of *F. albicollis*, this vocal blending did not promote interspecific hybridization. Similarly, in the contact zone of *Luscinia megarhynchos* and *L. luscinia*, although a high proportion of males (28–89%) produced blended songs or the songs of the other species, hybridization was relatively rare [82]. These results indicate that the interaction between vocalizations and gene flow may not be a simple cause-and-effect relationship playing out differently in different species and evolutionary contexts.

With technological advancements and as researchers use different analytical approaches to study the same system, there is a need to update or even redefine the existing theoretical models. In leaf warblers, an illustrative example of the impact of vocal divergence on reproductive isolation was the process of ring species formation within the *Phylloscopus trochiloides–plumbeitarsus* complex [105,106,107]. Song varied along the eastern and western slopes of the Tibetan Plateau. In the contact zone in central Siberia, the song of the two terminal populations showed significant differences, leading to reproductive isolation between the species. However, subsequent studies revealed mixed vocalizations and a gradient variation in certain acoustic parameters within the Siberian contact zone suggesting that vocal differences might not strictly impede breeding interactions between these populations, challenging the ring species model [108]. Peterson and Anamza (2017) strongly opposed the ring species hypothesis, arguing that there were multiple differentiated populations surrounding the Tibetan Plateau that did not conform to a pattern of geographic isolation by distance (IBD) [109]. Alcaide et al. (2014) have provided genomic evidence to support this, demonstrating genetic barriers within the ring distribution range and limited asymmetric gene flow between the two terminal populations in Siberia [110].

### 2.2. Plumage

Plumage, as a distinctive secondary sexual trait of birds, plays a significant role in various life history activities, including flight, predator evasion, mate selection, and social selection [111,112,113]. Traditionally, taxonomists use plumage divergence as a basis for classifying species and subspecies within bird lineages. The degree of divergence, particularly in patterns rather than simple color replacement, contributes to their taxonomic delimitation as separate species [114,115]. In the context of bird species, brighter and colorful plumage serves as an indicator of potential sexual and social partners [116,117]. Substantial plumage divergence is particularly crucial in SCZs, as it can lead to the accumulation of corresponding mate preferences and eventually hinder gene flow, facilitating the process of speciation [118]. 

In SCZs of closely related species, hybridization often leads to mixing and variation in plumage coloration. For instance, in a narrow SCZ of the white-throated magpie-jay (*Calocitta formosa*) and the black-throated magpie-jay (*Calocitta colliei*) in southern Jalisco, hybrids exhibit transitional throat plumage coloration ranging from black to white [119]. Comparative studies investigating both plumage and genomic divergence demonstrated a positive correlation between plumage divergence and reproductive isolation [120]. However, recent genetic studies have demonstrated that the relationship between plumage variation and genomic differentiation is more intricate than previously thought. Some systems show remarkable phenotypic divergence but little genomic divergence, contrasting with others that exhibit minimal or no discernible phenotypic divergence but significant genomic divergence [121]. Although these systems may represent exceptions, they underscore the complexity of understanding speciation and species delimitation based on these divergence metrics. In reviewing previous publications, the relationship between plumage divergence and genomic variation can be categorized into three main types (Figure 1b).

The first type is conspicuous plumage divergence accompanied by a mixed genetic structure. For example, individuals in the SCZ of the white-breasted antbird (*Rhegmatorhina hoffmannsi*) and Harlequin antbird (*R. berlepschi*) exhibited transitional throat and chest coloration from white to brown. Intermediate individuals (with ancestry coefficients of approximately 0.3–0.6) were found at the location of plumage color transition. Genomic clines revealed a single mitochondrial haplogroup and the introgression of 15 alleles with *R. hoffmannsi* ancestry into the *R. berlepschi* genomic background [122]. The second type is obvious plumage divergence despite limited genetic divergence. In cases where plumage divergence does not lead to reproductive isolation, genome homogenization and the emergence of phenotypic hybrids within the contact zone may occur. In the SCZ of two woodswallow species, continuous differentiation of plumage color was driven primarily by weak genomic differentiation [123]. In the SCZ of the Townsend’s warbler (*Setophaga townsendi*) and hermit warbler (*S. occidentalis*), more than half of the plumage variations could be explained by a 0.2 Mb genomic region containing three pigment accumulation genes (ASIP, EIF2S2, and RALY) [124]. The third type pertains to the variation in specific plumage traits resulting from distinct genetic variations at several key loci. In the SCZ of *Pogoniulus pusillus* and *P. chrysoconus*, the CYP2J19 gene was shown to be the main cause of forehead plumage color differences, leading to establishment of reproductive isolation [125]. Selection regarding plumage color may be enhanced in SCZs [126]. In hybrid populations of *Zosterops kulambangrae* and *Z. murphyi*, the size of the white eye ring was demonstrated to be critical in species recognition and reproductive isolation, which blocked gene flow through sexual selection [127]. The SCZ of the collared towhee (*Pipilo ocai*) and spotted towhee (*Pipilo maculatus*) in Central Mexico showed dramatic exhibited cline shifts in mtDNA and throat color, suggesting the possibility of sexual selection as a factor in differential introgression, while the contrasting cline shift in the green back color hints at a role for natural selection [67]. The fourth type is deep phylogenetic divergence which did not bring about plumage divergence. Differences in migration and breeding times might function as prezygotic isolation mechanisms. Two subspecies of *Riparia diluta* were genetically deeply differentiated but they only slightly varied in morphology. No signs of gene flow were detected along the SCZ of lowland south-eastern Chinese populations [128].

### 2.3. Beak Morphology

The beak is a critical trait that is involved in various ecological functions of birds. Due to the diverse diets and varied foraging behaviors exhibited by avian species, their beaks display substantial variability in terms of length, height, width, and curvature. This diversity indicates that selection pressure stands out as a key driving force behind the evolution of beak morphology [129]. Investigations into beak morphology have significantly contributed to our understanding of evolution and speciation in birds [130,131,132]. In classical taxonomy, beak morphology has long been employed as a crucial criterion for defining anatomical traits, foraging niches, and species classification [133].

On the contrary, the adaptability of the beak also enables birds to rapidly respond to ecological or environmental changes [134]. In particular, during secondary contact, variations in beak morphology may accelerate ecological niche differentiation among species, leading to rapid adaptive radiation [135,136]. A classic illustration of character displacement resulting from secondary contact is evident in Darwin’s finch system in the Galápagos Islands. Extensive secondary contact and interspecific hybridization have been confirmed among different species within this radiation [137]. Grant et al. [138] observed that, under the selection pressure imposed by the arid environmental conditions on Daphne Major Island, the hybrid population of the medium ground finch (*Geospiza fortis*) and common cactus finch (*G. scandens*) rapidly fixed a stable intermediate beak phenotype in terms of length and depth over a span of 30 years. This hybrid population also manifested significant differences in diet and foraging behavior compared to other species of ground finches on the island. Additionally, during the breeding season, they exclusively recognized the beak shape and song of members within their own population, refraining from mating with other species of ground finches on the island. This behavior accelerated the occurrence of hybrid speciation. In the genus *Pachyptila*, the sizes of beak differ between species and are characterized by incomplete reproductive isolation, leading to interspecific gene flow [139].

Although the diversification of beak types in Darwin’s finches has been extensively studied [137,138,140], beak types in the SCZ of other bird species have received comparatively little attention.

### 2.4. Migratory Behavior

Behavioral differences play a crucial role in the early stages of speciation by contributing to reproductive isolation [141]. In Wilson’s warblers (*Wilsonia pusilla*), the divergence in migratory behavior between western and eastern populations suggested a link to the speciation process [142]. In SCZs, the migratory behavior of hybrids may differ from that of their parent populations. Assortative mating by timing of arrival and selection against hybrids with intermediate migratory traits may maintain reproductive isolation between subspecies [143]. For example, genomic analysis of the yellow-breasted bunting (*Emberiza elegans*) revealed extensive gene flow between the southern resident and northern migratory populations, with hybrids displaying intermediate migratory behaviors [43]. In Sweden, the willow warbler (*Phylloscopus trochilus*) had an SCZ where southwestern and southeastern migratory subspecies intermingled. Most hybrids (76%) had migratory patterns similar to one of the parent subspecies, and epistatic interactions between two loci accounted for 74% of the variation, suggesting that they were not significantly affected by the costs associated with intermediate climate conditions [144]. Since behavioral isolation is often incomplete, postzygotic mechanisms, such as the selective elimination of hybrids with intermediate traits, also contribute significantly to avian diversification [145]. Additionally, migratory behavior in hybrid zones can appear random. In the Canadian Rockies, there is a narrow hybrid zone of *Setaphaga auduboni* and *S. coronata*, with the former migrating in the south-southwest direction and the latter in the southeast direction. Within this hybrid zone, there was no significant correlation between migratory direction and genetic background, leading to considerable variability in migratory patterns among individuals [146].

## 3. Spatiotemporal Dynamics

Although the movement of SCZs has previously been neglected [21,147], the temporal dynamics of SCZs are still considered one of the most popular topics for understanding how different evolutionary forces shape such situations [148]. Successive years of observations can provide insights into whether the dispersal between two species or the strength of the isolating barriers has changed [149]. In turn, this provides a better understanding of how past and current selection pressures affect the structure and distribution of the zone. The spatial changes in SCZs are attributed to both extrinsic and intrinsic factors. Climate, including the past and present conditions, has been shown to be the most critical factor in affecting SCZs [4,150]. Theoretically, the movement of SCZs could also be influenced by competition [151], asymmetrical migration [152], asymmetrical hybridization [153], dominance drive [154], and/or human activity [155].

Two widely accepted models that describe the maintenance and movement of SCZs are the tension zone model [156] and the environmental gradient model [157]. Endogenous selection for hybrids maintains the structure and balance in SCZs by continuously dispersing parents from the alien population in the tension zone model. In contrast, the environmental gradient model suggests that exogenous selection produces different degrees of fitness, and that the hybrid population disperses along environmental gradients. Both models are based on ecological stability, where changes in the environment lead to location shifts due to selection pressures [158]. According to the tension zone model, SCZs can keep moving until they reach regions where both parent species produce an equal rate of gene flow into the SCZ [159,160]. The movement of SCZs in the environmental gradient selection model depends on the fitness gradient and habitat changes in the SCZ [161]. If hybrids show higher fitness in the intermediate environment than that of the parent population, the SCZ will expand [162].

The movements of SCZs can be theoretically predicted, but it is difficult to directly prove such events through continuous experimental observations; in addition, it is difficult to obtain field samples that are equivalent in the long term [149]. Evidence for moving SCZs can be obtained directly through repeated sampling over time or indirectly through the detection of asymmetric patterns of introgression [163]. Analysis of allele frequency changes over decades can provide unequivocal evidence of contemporary spatial shifting of an SCZ under recent variable landscapes and climate changes [164,165,166]. However, repeated samples over long periods are difficult to collect because of location shifts and the persistence of research programs, so most moving SCZs have been detected by introgression analysis [163]. Most of the SCZs were surveyed for less than 5 years before the twenty-first century, but population densities and environmental gradients changed rather slowly [21]. However, many recent studies have provided long-term empirical evidence of SCZ movements [148,167,168]. Previous studies have shown that the movement of SCZs may be more common than previously thought [22,148,149,169].

Recent follow-up studies on some classic systems have yielded valuable insights into the temporal dynamics of SCZs. The maintenance of clines is achieved through a delicate balance between gene flow and barriers, selection and dispersal, and divergence within each population [170]. In Puerto Rico, dispersal and selection balanced a geographically stable SCZ for *Sphaerodactylus nicholsi* and *Sphaerodactylus townsendi* [171]. Occasionally, a moving SCZ may stabilize due to the loss of a suitable habitat. Location shifts represent the most common pattern for moving SCZs. For instance, genomic comparisons indicated that cold winter temperatures drove the SCZ of the black-capped chickadee (*Poecile atricapillus*) and the Carolina chickadee (*P. carolinensis*) in southeastern Pennsylvania to move northward between two sampling periods, from 2000 to 2002 and from 2010 to 2012 [150]. The speed of SCZ movement has been demonstrated to be related to temperature changes [172]. An SCZ of tanagers in the genus *Ramphocelus* (Aves, Thraupidae) located in southwestern Colombia has shifted eastward and to higher elevations, and possibly narrowing in recent decades [167]. Conversely, the SCZ of yellow-shafted and red-shafted flickers in the Great Plains exhibited a ~73 km westward shift in the center toward the range of the red-shafted flicker with no associated changes in width over the sampling periods (1955–1957 to 2016–2018) [173]. Furthermore, SCZ fragmentation has occurred, potentially leading to the disappearance of SCZs through fission or species displacements. The extinction of the SCZ of Townsend’s warblers and hermit warblers was attributed to movements driven by interspecific competition [174]. Competitive displacement of hermit warblers by Townsend’s warblers was predicted to ultimately result in the disappearance of the SCZ. The SCZ of the greater spotted eagle (*Aquila clanga*) and lesser spotted eagle (*Aquila pomarine*) is another well-studied system for temporal dynamics [175,176]. In most regions, hybridization was common and took place predominantly between *A. pomarina* males and *A. clanga* females. In the course of 16 years of genetic monitoring of a mixed population in Estonia, we observed the abandonment of *A. clanga* breeding territories and the replacement of *A. clanga* pairs by *A. pomarina*, whereby on several occasions, hybridization was an intermediate step before the disappearance of *A. clanga*. This posed an additional threat for the vulnerable *A. clanga* and might contribute to the extinction of its populations [19,177]. 

## 4. Perspectives

The patterns and mechanisms of speciation are central questions in the field of evolutionary biology [178,179,180]. Interspecific hybridization caused by secondary contact has led to multiple consequences for the original differentiated population, which is an important cause of species diversification. SCZs mostly exist in biodiversity hotpots under some natural conditions [181,182,183,184] and are also of great significance in biodiversity conservation [185,186]. Significant progress has been achieved in the study of SCZs over the past decade. By summarizing the previous publications on SCZs, we have demonstrated the variations in three avian phenotypical traits in SCZs as examples and summarized the different patterns of spatiotemporal dynamics. These variations may eventually cause different evolutionary consequences for avian population over time.

Hybrid speciation in SCZs occurs when the hybrids occupy their unique niche and reproductive isolation develops between parental populations [187]. Several bird species have been reported to originate from hybrid speciation, such as Italian sparrows (*Passer italiae*) [188,189], golden-crowned manakins (*Lepidothrix vilasboasi*) [16], and Hawaiian ducks (*Anas wyvilliana*) [190]. The Italian sparrow was first hypothesized to have a hybrid origin because of the phenotypic similarity of hybrids of the house sparrow (*P. domesticus*) and the Spanish sparrow (*P. hispaniolensis*) [191]. Reinforcement refers to the scenario in which natural selection acts against hybrids with low fitness, creating gene flow barriers between sympatric heterospecific populations and enhancing reproductive isolation [192]. This process contributes to the establishment of reproductive isolation between recently diverged populations in SCZs, resulting in two distinct species [193,194]. The speciation of the pied flycatcher (*Ficedula hypoleuca*) and the collared flycatcher (*Ficedula albicollis*) are among the few proposed examples of the process of reinforcement of premating isolation that are supported by compelling evidence. They are also characterized as having strong intrinsic postzygotic barriers (female hybrid sterility), yet the two species are very similar ecologically. Secondary contact may cause population fusions and even reverse speciation by reconstructing gene flow between highly differentiated and divergent species [195]. Fusion between mtDNA lineages was found in the common raven (*Corvus corax*) [196]. In SCZs, sexual selection through male competition [98] and ecological shifts are associated with reverse speciation [197]. Hybridization in SCZs may eventually cause extinction through two main mechanisms. The first mechanism is outbreeding depression [198]. If the fitness of hybrids is strongly reduced in SCZs, the populations of the parental species might decline because of the wasted energy used for breeding. This mechanism is named demographic swamping [199]. If the fitness of hybrids is strongly increased in SCZs, the populations of one or both parental species may be replaced by hybrids, which is termed genetic swamping [18]. The range of Townsend’s warblers is expanding and encroaching on the ecological niches of hermit warblers, which brings about competitive displacement [174]. This process may eventually cause hermit warblers to go extinct. 

Several questions remain unanswered in so far as we reviewed: (1) Stable SCZs have long been used to study the factors affecting reproductive isolation [200], but the degree of divergence required for complete reproductive isolation varies widely between taxa, which makes the consequence of secondary contact hard to predict [201]. A comprehensive study comparing different groups with varying degrees of divergence may provide an answer. (2) SCZs have complex patterns of movement, which require an accurate prediction model for predicting future movements under climate change and anthropogenic disturbances. (3) At present, there is no clear theory to explain the relationship between phenotypic divergence and the degree of genomic divergence in SCZs. Current studies usually start with individual cases, which have a greater degree of randomness. (4) Even for the same pair of interacting species, there might be differences in phenotypic variations, genetic variations, and compositions between different contact zones, and differences in the proportion of hybrid individuals in different hybrid regions of the same species. In a relatively newly formed SCZ in Sweden, approximately 2–7% of individuals are hybrid individuals [202], while in an earlier-formed contact zone in western Russia, hybrid individuals are relatively rare (<1% [80]). The comparison of multiple SCZs may require more research and discussion. (5) Influenced by historical climatic and geographical events, some regions may serve as shared secondary contact zones for multiple species. Future research on these areas is crucial for understanding the mechanisms behind the formation of biodiversity hotspots. (6) Similar phenotypic divergence patterns may play comparable roles in reproductive isolation across multiple secondary contact zones. Comparing the genomic bases of these phenotypic divergences could help identify the key genes or structural variants driving avian speciation.

Research related to SCZs has also made progress in groups other than birds. A variety of natural SCZs are formed by postglacial secondary contact from glacial refugium expansion [203], for instance, gray foxes in America [204] and grasshoppers from the North American Rockies [205]. Many SCZs have also been monitored and sampled continuously over decades. For example, the genetic and acoustic structures of an SCZ of two species in the brown tree frog (*L. ewingii*) complex illustrated the stability of the SCZ over 40 years [148]. Some research findings from avian studies are worth referencing, for example, evolutionary rescue. Convincing evidence for evolutionary rescue comes from the introgression that occurred in the SCZ of medaka (*Fundulus grandis*) and Canadian medaka (*F. heteroclitus*) [206]. Under severe pollution, medaka [207] had obtained a deletion of the aryl hydrocarbon receptor (AHR) gene from Canadian medaka through secondary contact and adaptive introgression, which hindered the induction of aromatic hydrocarbons in medaka body signal transduction, thereby enhancing its resistance to environmental pollution [206].

The current research on the genetic structure of contact zones is mostly based on resequencing data. Third-generation genome sequencing technologies, such as those from Pacific Biosciences (PacBio) and Oxford Nanopore Technologies (ONT), combined with complementary methods like Hi-C, have been increasingly employed in SCZ studies, offering enhanced accuracy and high-quality datasets [208,209,210]. Combining different sequencing methods has become a common strategy to obtain more precise results, highlighting the promising future of SCZ research [209,211,212]. With the popularization of third-generation sequencing technology and the development of genome-wide assembly methods, it is hoped that a new analytical method and analysis paradigm will be established. We believe that more detailed studies on population structures, ancestral distributions, and niche innovations will facilitate the understanding of the formation and movement of SCZs.

## 5. Conclusions

The relationship between trait variation and genomic differentiation in avian SCZs is complex and varies across different study systems. This variation may be attributed to differences in divergence times and stages among the study subjects, as well as differences in the rate of trait fixation among taxa. Due to interspecies interactions, climate change, and human-induced disturbances, different SCZs may evolve along distinct evolutionary trajectories. From the perspective of species diversity, SCZs significantly influence avian species diversity by enhancing genetic variation, facilitating the emergence of novel phenotypic traits and promoting gene flow, thereby improving local adaptation and increasing genetic diversity, which helps birds adapt to changing environmental conditions, enhancing their survival and reproductive success. Furthermore, SCZs may serve as hotspots for speciation, where species boundaries become less distinct, accelerating the formation of new species and lineage divergence. Conversely, SCZs can negatively impact avian diversity by diluting the genetic purity of native species, which can undermine their uniqueness and adaptability, and may also contribute to extinction events through interspecies competition. Thus, the effects of hybrid zones are complex and multifaceted. Investigating the dynamics of hybrid zones and their impact on bird species diversity is essential for a comprehensive understanding of avian evolution and the mechanisms underlying biodiversity formation. Detailed research into these zones is crucial for elucidating their intricate role in biodiversity conservation.
